# The PHACS SMARTT Study: Assessment of the Safety of *In Utero* Exposure to Antiretroviral Drugs

**DOI:** 10.3389/fimmu.2016.00199

**Published:** 2016-05-23

**Authors:** Russell B. Van Dyke, Ellen Gould Chadwick, Rohan Hazra, Paige L. Williams, George R. Seage

**Affiliations:** ^1^Department of Pediatrics, Tulane University School of Medicine, New Orleans, LA, USA; ^2^Department of Pediatrics, Feinberg School of Medicine, Northwestern University (NUFSM), Chicago, IL, USA; ^3^Eunice Kennedy Shriver National Institute of Child Health and Human Development, Bethesda, MD, USA; ^4^Department of Biostatistics, Harvard T.H. Chan School of Public Health, Boston, MA, USA; ^5^Department of Epidemiology, Harvard T.H. Chan School of Public Health, Boston, MA, USA

**Keywords:** HIV exposure, *in utero*, children, safety, toxicity, antiretroviral drugs, infant, newborn

## Abstract

The Surveillance Monitoring for ART Toxicities (SMARTT) cohort of the Pediatric HIV/AIDS Cohort Study includes over 3,500 HIV-exposed but uninfected infants and children at 22 sites in the US, including Puerto Rico. The goal of the study is to determine the safety of *in utero* exposure to antiretrovirals (ARVs) and to estimate the incidence of adverse events. Domains being assessed include metabolic, growth and development, cardiac, neurological, neurodevelopmental (ND), behavior, language, and hearing. SMARTT employs an innovative trigger-based design as an efficient means to identify and evaluate adverse events. Participants who met a predefined clinical or laboratory threshold (trigger) undergo additional evaluations to define their case status. After adjusting for birth cohort and other factors, there was no significant increase in the likelihood of meeting overall case status (case in any domain) with exposure to combination ARVs (cARVs), any ARV class, or any specific ARV. However, several individual ARVs were significantly associated with case status in individual domains, including zidovudine for a metabolic case, first trimester stavudine for a language case, and didanosine plus stavudine for a ND case. We found an increased rate of preterm birth with first trimester exposure to protease inhibitor-based cARV. Although there was no overall increase in congenital anomalies with first trimester cARV, a significant increase was seen with exposure to atazanavir, ritonavir, and didanosine plus stavudine. Tenofovir exposure was associated with significantly lower mean whole-body bone mineral content in the newborn period and a lower length and head circumference at 1 year of age. With ND testing at 1 year of age, specific ARVs (atazanavir, ritonavir-boosted lopinavir, nelfinavir, and tenofovir) were associated with lower performance, although all groups were within the normal range. No ARVs or classes were associated with lower performance between 5 and 13 years of age. Atazanavir and saquinavir exposure were associated with late language emergence at 1 year, but not at 2 years of age. The results of the SMARTT study are generally reassuring, with little evidence for serious adverse events resulting from *in utero* ARV exposure. However, several findings of concern warrant further evaluation, and new ARVs used in pregnancy need to be evaluated.

Antiretroviral (ARV) therapy during pregnancy has dramatically reduced the rate of mother-to-child transmission of HIV, which is currently 2% or less in the US (1). However, the number of HIV-infected pregnant women has not decreased, resulting in a large number of infants who are not HIV infected but who were exposed, *in utero*, to ARVs. Toxicities from *in utero* exposure to ARVs, including mitochondrial toxicity, remain a major concern (2). Nucleoside reverse transcriptase inhibitors (NRTIs) induce mitochondrial dysfunction by inhibiting replication of mitochondrial gamma DNA polymerase, leading to mitochondrial DNA depletion, mutations, and dysfunction (3). Some studies have identified clinical symptoms, including lactic acidosis, cardiomyopathy, and neurological abnormalities, suggesting mitochondrial dysfunction, in a small proportion of exposed infants (4–6).

The Pediatric HIV/AIDS Cohort Study (PHACS) includes a network of 22 clinical sites in the US and Puerto Rico. It conducts three longitudinal cohort studies of children born to HIV-infected mothers: (1) the Surveillance Monitoring for ART Toxicities (SMARTT) study of HIV-exposed but uninfected (HEU) infants and children, (2) the adolescent master protocol (AMP) study of perinatally HIV-infected children and adolescents, and (3) the AMP up protocol which follows AMP subjects into young adulthood once they reach 18 years of age. AMP and AMP up also include a comparison group of HEU. The objective of SMARTT is to determine the safety of *in utero* exposure to ARVs among HEU children and to estimate the incidence of adverse events. Domains being assessed include metabolic, growth and development, cardiac, neurological, neurodevelopmental (ND), behavior, language, and hearing. Study visits are conducted annually until 5 years of age and then every other year, with specified clinical and laboratory evaluations. In addition, we enrolled a comparison group of 239 HIV-unexposed and uninfected (HUU) children of similar sociodemographic background to that of the SMARTT subjects at 1, 3, 5, and 9 years of age. These participants had a single evaluation and were not followed longitudinally.

The SMARTT study opened to enrollment in 2007 at 22 sites and includes 2 cohorts of HIV-infected mothers and their HEU children. The Static cohort enrolled 1,240 children less than 12 years of age at enrollment and closed to further enrollment in 2009. Seventy-seven percent of participants remain on study with a median age of 11.0 years and a median duration of follow-up of 7.3 years. The dynamic cohort remains open to enrollment. As of March 2016, it includes over 2,300 mother–infant pairs who were enrolled during gestation or within 72 h of birth. Approximately 250 mother–infant pairs are enrolled each year. Eighty-five percent of dynamic cohort participants remain on study with median age of 3.2 years and a median duration of follow-up of 3.7 years. Overall, 48% of the SMARTT participants are females, 66% Black, and 33% Hispanic. Retention has been excellent with 85% of static and 80% of dynamic participants remaining on study at 6 years. For additional characteristics of the SMARTT cohort, see Williams et al. (7).

There are a number of strengths of SMARTT. A complete history of *in utero* ARV exposure (8), coupled with longitudinal assessments from birth in the dynamic cohort, allows for careful consideration of various windows of exposure and how they may affect specific outcomes. Trigger-based surveillance is an efficient means to identify adverse events (9). The study addresses an extended spectrum of outcomes including epigenetic changes, alterations in mitochondrial DNA and intermediary metabolism, and bone density in infants. It includes novel measures of *in utero* exposure to ARVs, alcohol, and recreational drugs using meconium and hair (10, 11). It allows for nested, more intensive substudies, such as those addressing maternal nutrition and tenofovir exposure of the fetus. Untangling the effect of individual ARVs is challenging, since most infected women take multiple ARVs during pregnancy. Assembling a comparison group of HEU children who were not exposed to ARVs *in utero* is not feasible in the US. Thus, most analyses compare subgroups of participants who were exposed or not exposed to different ARVs or ARV classes, considering the trimester of exposure or duration of exposure.

## Trigger-Based Design to Identify Adverse Event

In SMARTT, we employ an innovative trigger-based design as an efficient means to identify and evaluate adverse events, to estimate adverse event rates, and to test for association with *in utero* and perinatal ARV exposures (9). Participants who meet a predefined clinical or laboratory threshold or “trigger” undergo additional specified evaluations to determine if they meet a predefined adverse event “case” definition. Most cases are identified using a computerized algorithm, eliminating the need for individual reviews. A SMARTT Review panel, comprising clinicians, biostatisticians, and epidemiologists, oversees the identification of cases and does individual reviews of participants meeting a trigger based on abnormal laboratory values and neurologic diagnoses. The specific triggers evaluated are shown in Table S1 of Supplementary Material. The domains considered were selected because they are known or suspected to result from mitochondrial dysfunction or other ARV-associated toxicities.

**Figure 1:**
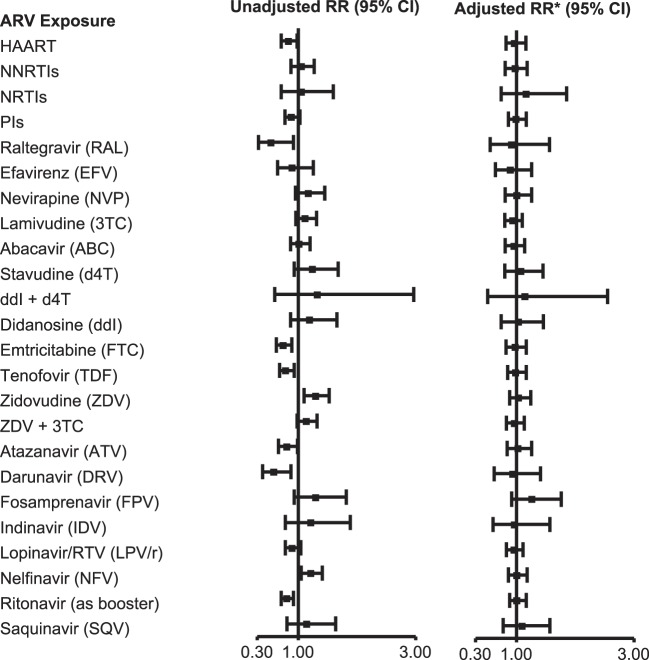
**Association of overall adverse event case status with *in utero* antiretroviral (ARV) exposures**. *Adjusted model includes Black race or Puerto Rican origin, low caregiver education (<high school), first trimester maternal tobacco use, and birth cohort (≥2010 vs. <2010). Abbreviations: RR, relative risk; HAART; cARV, combination antiretrovirals; NNRTI, non-nucleoside reverse transcriptase inhibitor; NRTI, nucleoside or nucleotide reverse transcriptase inhibitor; PI, protease inhibitor. From Ref. (7), used with permission.

Among 2,680 SMARTT participants enrolled on December 31, 2012, 48% met a trigger and 25% met case status in one or more domains (7). After adjusting for birth cohort and other factors, there was no significant increase in the likelihood of meeting overall case status (case in any domain) with exposure to combination ARVs (cARV) or any ARV class (Figure 1). No single ARV was associated with an increased risk of overall case status on adjusted analysis, although several were significant in the unadjusted analysis. However, several ARVs were significantly associated with case status in individual domains. Maternal zidovudine use was associated with meeting the metabolic case definition [adjusted relative risk (aRR) 1.69, 95% CI 1.08, 2.64, *p* = 0.022]. First trimester stavudine was associated with meeting the language case definition (aRR 2.06, 95% CI 1.04, 4.09, *p* = 0.038), and didanosine plus stavudine use was associated with meeting the ND case definition (aRR 12.4, 95% CI 5.29, 29.1, *p* = 0.001). A number of exposures were protective of meeting case status, including, for metabolic case status, cARV, protease inhibitors (PIs), emtricitabine, ritonavir-boosted lopinavir, and ritonavir, and for ND case status, cARV.

## Pregnancy Outcomes

**Figure 2:**
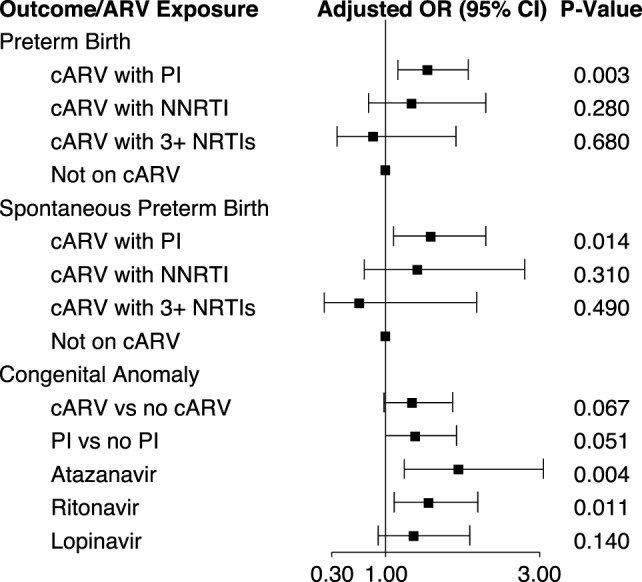
**First trimester antiretroviral exposure and pregnancy and infant outcomes (15, 16)**. Abbreviations: cARV, combination antiretrovirals; PI, protease inhibitor; NNRTI, non-nucleoside reverse transcriptase inhibitor; NRTI, nucleoside or nucleotide reverse transcriptase inhibitor.

Maternal PI-based cARV during pregnancy has been associated with an increased rate of prematurity and low birth weight (12–14). In an analysis of 1,864 singleton births in the dynamic cohort, 18.6% of infants were preterm (<37 weeks gestation) and 7.3% small for gestational age (weight <10 percentile for gestational age). Eighty-nine percent of the mothers took cARV during their pregnancy. We found an increased risk of preterm birth [odds ratio (OR) 1.55, 95% CI 1.16, 2.07] and spontaneous preterm birth (OR 1.59, 95% CI 1.10, 2.30) with first trimester exposure to PI-based cARV, but not with NNRTI-based cARV or ≥3 NNRTIs, compared to no first trimester ARV use (15) (Figure 2). cARV use later in pregnancy was not associated with an increased risk. No significant increase in the rate of small for gestational age infants was observed with any regimen.

We investigated the association of birth defects with first trimester ARV exposure among 2,580 infants in both the static and dynamic cohorts, which included a review of individual defects by the study team. The overall prevalence of birth defects was 6.78 per 100 live births (95% CI 5.85, 7.82), higher than the general population estimate of 2.67% of the Metropolitan Atlanta Congenital Defects Program (16). It is also higher than that reported by the International Antiretroviral Pregnancy Registry (2.9%) and the Women and Infants Transmission Study (3.56%) for first trimester ARV exposure (17). There was a significant increase in the prevalence of birth defects over the course of the study. Among all reported birth defects, the most common were musculoskeletal (29.8%) and cardiovascular (23.1%). In unadjusted analysis, the occurrence of birth defects was significantly increased with first trimester cARV exposure (OR 1.44, 95% CI 1.05, 1.97) and first trimester PI exposure (OR 1.51, 95% CI 1.10, 2.07). However, when adjusted for birth cohort, CD4 during early pregnancy and prematurity, the risks remained similar (OR 1.35 and 1.39, respectively) but were no longer significant (Figure 2). Among individual ARVs or ARV combinations, first trimester exposure to the PIs atazanavir (OR 1.95, 95% CI 1.24, 3.05) and ritonavir (as a boosting agent) (OR 1.56, 95% CI 1.11, 2.20) and the combination of didanosine plus stavudine (OR 8.19, 95% CI 1.53, 43.4) were associated with a significantly increased rate of birth defects in adjusted analysis. Specific congenital defects that were significantly associated with specific ARV exposures include male genital (zidovudine and lamivudine), musculoskeletal (atazanavir, ritonavir, and didanosine plus stavudine), cardiovascular (atazanavir and ritonavir), and skin (atazanavir).

## Lactic Acidosis

An elevated blood lactate level is useful in screening for mitochondrial dysfunction. Because of the difficulty in obtaining an appropriate venous blood sample in children, we utilized the point-of-care (POC) Lactate Pro device to measure capillary blood lactate concentrations annually in SMARTT (18). Among 1,934 participants at a mean age of 3.0 years, 3.4% had a POC lactate >3 mmol/L. The mean lactate level decreased as the children aged. The prevalence of elevated lactate following *in utero* exposure did not differ by ARV class but was significantly higher in those exposed to emtricitabine, efavirenz, cocaine, or opiates. No clinical findings were associated with an elevated lactate, but further analyses are planned.

## Growth

Reports of the effects of *in utero* ARV exposures on growth of HEU children are limited and have not revealed consistent patterns (19–21). Rapid weight gain leading to obesity and associated metabolic problems is increasing among children in the US. SMARTT participants were smaller than US reference standards at birth, with a mean weight *z*-score of −0.56 (SD 0.85) and length *z*-score of −0.16 (SD1.0) (22). Nineteen percent were small for gestational age. By 2 years of age, their mean weight had caught up with US norms and continued to increase with age.

Use of tenofovir has been associated with loss of bone mineral density in adults (23) and children (24) and concerns about bone growth in non-human primates (25). *In utero* tenofovir exposure and low maternal vitamin D are associated with decreased fetal bone accrual and possible future fracture risk (26–28). Currently, 70% of the pregnant mothers in SMARTT are receiving tenofovir during their pregnancy. In order to define the effect of *in utero* tenofovir exposure on newborn bone mineral content, we performed dual-energy X-ray absorptiometry (DXA) scans at 2–3 weeks of age on 74 tenofovir-exposed and 69 unexposed infants (27). While the mean weight and length of the two groups at birth were similar, we found a 12% lower mean whole-body bone mineral content in those exposed to tenofovir (56.0 vs. 63.8 g, *p* = 0.002), which persisted when adjusted for maternal age, smoking, HIV disease status and infant gestational age, sex, race, and length (*p* = 0.013). We are currently planning to repeat the DXA scans among these children at an older age to see if this finding persists.

We compared the growth at birth and 1 year of age of 449 infants exposed *in utero* to cARV with tenofovir to 1,156 exposed to cARV without tenofovir. At birth, their mean weight, length, and head circumference were below US norms but similar in the two groups (*z*-scores: −0.63 and −0.66, −0.25 and −0.16, and −0.66 and −0.65, respectively) (29). There was no increased risk of low birth weight or being small for gestational age with tenofovir exposure. However, at 1 year of age, those with *in utero* tenofovir exposure had a slightly lower mean length and head circumference [difference in adjusted mean *z* scores: −0.14 (*p* = 0.04) and −0.25 (*p* = 0.02), respectively], but their weights were similar.

## Cardiac Function

Mitochondrial dysfunction frequently affects the heart, and subclinical changes in left ventricular structure and function have been reported in HEU children with *in utero* ARV exposure (30). Thus, we performed echocardiograms on 417 HEU and 98 HUU children between 2 and 7 years of age (31). We found no significant difference in measures of myocardial structure or function between the two groups. Among the HEU participants, we observed subclinical differences in measures of left ventricular structure and function with first trimester exposure to cARV and to specific ARVs. The left ventricular stress-velocity index (myocardial contractility) was significantly decreased with exposure to cARV, tenofovir, emtricitabine, and ritonavir-boosted lopinavir, whereas the left ventricular end-diastolic posterior wall thickness was significantly elevated among those exposed to cARV, zidovudine, lamivudine, nevirapine, and atazanavir. These subclinical findings suggest injury to the myocardium and highlight the need for longitudinal cardiac follow-up to assess long-term cardiac risk. About half of the HEU children in SMARTT had elevated levels of at least one cardiac or inflammatory biomarker, suggesting that chronic inflammation may contribute to these cardiac finding and may help to identify HEU children who need further cardiac evaluation (32).

## Neurocognitive and Language Functioning

Findings of cognitive deficits and structural and functional changes in the brains of animals and humans with ARV exposure support the need for continued study of brain and neurocognitive development, as the brain is heavily dependent upon mitochondrial integrity and is rapidly developing during *in utero* ARV exposure (33–37). HEU youth are at increased risk for poor ND outcomes and while specific ARVs or classes have not been clearly implicated, concerns persist (38–41). Language acquisition is essential for social, academic, and long-term employment.

We evaluated 374 HEU and 49 HUU children from a similar background between 9 and 15 months of age using the Bayley-III assessment (41). Eighty-four percent of the HEU group had been exposed to cARV *in utero*. There were no significant differences between HEU and HUU infants or between cARV-exposed and unexposed HEU children in any of the five Bayley-III domain scores. In four of the five domains, the cARV-exposed group had a higher (although not significantly) adjusted mean score than the cARV-unexposed group. A number of significant differences were seen in the HEU groups with exposure to specific ARVs. Atazanavir exposure was associated with significantly poorer language performance, ritonavir-boosted lopinavir was associated with lower adaptive behavior performance, nelfinavir was associated with lower cognitive performance, and tenofovir was associated with lower social–emotional performance. In contrast, lamivudine exposure was associated with significantly higher social–emotional performance and ritonavir-boosted lopinavir was associated higher language performance. However, for all of these comparisons, both groups had median scores within the normal range.

For participants between 5 and 13 years of age, assessments included the Wechsler Preschool and Primary Scale of Intelligence-III (WPPSI-III) (age 5), the Wechsler Abbreviated Scale of Intelligence (ages 7, 9, 11, and 13) for cognitive status, and the Wechsler Abbreviated Individual Achievement Test (ages 7, 9, 11, and 13) for academic performance (42). We found no association between *in utero* exposure to any ARV regimen or class and any cognitive or academic outcome. No individual ARV was significantly associated with lower cognitive or academic scores in unadjusted and adjusted analyses. Exposure to tenofovir was associated with a significantly higher WPPSI-III score (100.8 vs. 96.1, *p* = 0.03).

Language emergence was evaluated at 1 year of age with the MacArthur–Bates Communicative Development Inventory (CDI) and at 2 years with the ages and stages questionnaire (ASQ). Late language emergence was defined as <10 percentile for any of four domains of the CDI and ≥1 SD below the age-specific normal value for the ASQ. Late language emergence was found in 26% of 1-year-old HEU children and was independently associated with male sex, maternal CD4 <25%, viral load >400 copies/mL during late pregnancy, and caregiver IQ <85 (43). At 2 years of age, late language emergence was present in 23% and was independently associated with male sex and having a caregiver with limitations in activities. There was no significant association of late language emergence at either 1 or 2 years of age with maternal use of cARV, a PI, or an NNRTI. However, at 1 year, but not at 2 years, late language emergence was significantly and independently more likely among children whose mothers took atazanavir (OR 1.83, 95% CI 1.10, 3.04, *p* = 0.02) or saquinavir (OR 2.72, 95% CI 1.09, 6.91, *p* = 0.03) during the pregnancy. In addition, use of cARV by the newborn for prophylaxis, vs. zidovudine alone, was independently associated with late language emergence at 1, but not 2 years of age (OR 3.07, 95% CI 1.18, 7.97, *p* = 0.02). Among AMP participants, at a mean age of 12.0 years, we demonstrated that both HIV-infected and HEU children were at high risk for language impairment, although the risk was similar in the two groups (44).

## Conclusion

The results of the SMARTT study to date are generally reassuring, with little evidence for serious adverse events resulting from *in utero* ARV exposure. However, there are several findings of concern that warrant further evaluation. The increased rate of premature delivery and selected birth defects with certain ARV exposures informs the choice of ARV for use during pregnancy. Atazanavir exposure is associated with lower language achievement at 1 year and a twofold higher risk of congenital anomalies (Figure 1). Atazanavir is known to increase unconjugated bilirubin levels in the blood, raising the concern that fetal bilirubin exposure resulting from transplacental passage of bilirubin could contribute to these findings. The association of tenofovir exposure with decreased newborn bone mineral content and reduced growth at 1 year of age are predicted by prior studies and warrants further study to see if these findings persist as the children’s age. Likewise, it is important to determine if the subclinical cardiac abnormalities predict future premature heart disease. Finally, it is important to study new ARVs as pregnant women adopt their use.

## Author Contributions

All authors meet all of the following criteria: substantial contributions to the conception or design of the work; acquisition, analysis, or interpretation of data for the work; drafting the work or revising it critically for important intellectual content; final approval of the version to be published; and agreement to be accountable for all aspects of the work in ensuring that questions related to the accuracy or integrity of any part of the work are appropriately investigated and resolved.

## Conflict of Interest Statement

RD: no conflicts. His institution received grant funds from the NIH to conduct the PHACS study. EC: no conflicts. Her institution received grant funds from the NIH to conduct the PHACS study. RH: no conflicts. He is employed by the NIH NICHD. PW: no conflicts. Her institution received grant funds from the NIH to conduct the PHACS study. GS: no conflicts. His institution received grant funds from the NIH to conduct the PHACS study.
