# Supplementary material for: The PHACS SMARTT Study: Assessment of the Safety of In Utero Exposure to Antiretroviral Drugs
Source: Front Immunol. 2016 May 23;7:199. doi: 10.3389/fimmu.2016.00199 (PMC4876360; doi:10.3389/fimmu.2016.00199)
Supplement: Supplementary file 1 [file Table_1.docx]

**Supplemental Table 1. Definition of Domain-Specific Triggers in SMARTT [1]**

- Growth: Low height or weight (<3^rd^ percentile for age), low growth rates, low mid-upper arm circumference or triceps skinfold (<5^th^ percentile for age)
- Metabolic: High body mass index (>95^th^ percentile for age)
- Lactate: Elevated point-of-care blood lactate (>3 mmol/L), confirmed on repeat testing
- Neurologic: Neurologic diagnosis (e.g. microcephaly, seizure)
- Language: Impairment (scores >2 SDs below age-specific normal values)
- Neurodevelopment: Impairment (scores >2 SDs below age-specific normal values)
- Lab: Abnormality chemistry or hematology value, confirmed on repeat testing*
- Hearing Impairment: Sensorineural or mixed hearing loss

* Grade 3 or higher based on NIH Division of AIDS Toxicity Tables. [2]

1. Williams PL, Hazra R, Van Dyke RB, et al. Antiretroviral exposure during pregnancy and adverse outcomes in HIV-exposed uninfected infants and children using a trigger-based design. Aids **2016**; 30(1): 133-44.

2. U.S. Department of Health and Human Services, National Institutes of Health, National Institute of Allergy and Infectious Diseases, Division of AIDS.  Division of AIDS (DAIDS) Table for Grading the Severity of Adult and Pediatric Adverse Events, Version 2.0. Available at: <http://rsc.tech-res.com/safetyandpharmacovigilance/gradingtables.aspx>. Accessed May 13, 2016.
